# Dorland’s Pocket Medical Dictionary

**Published:** 1914-03-15

**Authors:** 


DORLAND'S POCKET MEDICAL DICTIONARY. The eighth Edition of this work comes to us in a revised and much enlarged form, several hundred additional terms have been added. Necessarily most of these are due to the increasing amount of scientific research, which is continually increasing medical nomenclature that expression may be given to modern attainments. In this new nomenclature is recorded the periodic literature one needs, an up to the minute dictionary to read intelligibly. The pocket medical dictionary was originally designed for medical students, but it is because of its convenient size and terse definitions a most convenient and useful work tool for the busy practitioner or literary student. It is almost impracticable to critically review such a book. We have tested it for a number of terms and are agreeably surprised to find them all well defined. The typography is all that any one could desire, and is in keeping with the well known standards of the W. B. Saunders Co. its publishers.

